# Pharmacological Effects of *Lactuca serriola* L. in Experimental Model of Gastrointestinal, Respiratory, and Vascular Ailments

**DOI:** 10.1155/2013/304394

**Published:** 2013-04-04

**Authors:** Khalid Hussain Janbaz, Muhammad Farhaj Latif, Fatima Saqib, Imran Imran, M. Zia-Ul-Haq, Vincenzo De Feo

**Affiliations:** ^1^Faculty of Pharmacy, Bahauddin Zakariya University, Multan 60800, Pakistan; ^2^Department of Pharmacognosy, University of Karachi, Karachi 75270, Pakistan; ^3^Department of Pharmacy, University of Salerno, Via Ponte don Melillo, 84084 Fisciano, Italy

## Abstract

*Lactuca serriola* L. has traditionally been used in folkloric medicine to manage respiratory, gastrointestinal, and multiple other ailments. The present study was undertaken to explore the effect of methanol extract of *L. serriola* on isolated rabbit tissue preparations, that is, jejunum, trachea, and aorta in an attempt to validate its folkloric use in traditional medicine for gastrointestinal, respiratory, and vascular ailments. The application of the methanol extract to isolated rabbit jejunum preparations exhibited concentration-dependent spasmogenic effect (0.03 to 3.0 mg/mL), but interestingly further increase in concentration (5.0 mg/mL) resulted in complete spasmolytic effect. The pretreatment of the tissue preparations with atropine (0.1 **μ**M) caused the suppression of the contractile response. Moreover, the same extract also caused relaxation of K^+^-(80 mM) induced spastic contractions of isolated rabbit jejunum preparations (5.0 mg/mL) and shifted the Ca^++^ dose response curves towards right at concentration range of 0.3–1.0 mg/mL. Similarly, the extract application to isolated rabbit tracheal preparations relaxed the carbachol-(1 **μ**M) induced (0.3–1.0 mg/mL) as well as K^+^-(80 mM) induced contractions (3.0 mg/mL). Furthermore, it relaxed the phenylephrine (1 **μ**M)-induced contractions in isolated rabbit aorta preparations (3.0 mg/mL) and K^+^ (80 mM)-induced contractions (1.0 mg/mL). These effects were found comparable to that of dicyclomine, as an antagonist of muscarinic receptors as well as a possible Ca^++^ channel blocker. The previously mentioned findings may partially justify the folkloric use of *Lactuca serriola* in the management of conditions pertaining to spasm of intestine, bronchioles, and vasculature.

## 1. Introduction

*Lactuca serriola* L. (Compositae) is an herbaceous species, known by several vernacular names, that is, Prickly lettuce, Jagged lettuce, Kahu and Khas [1]. It is native to Himalaya, Siberia, and Atlantic areas [2] but cultivated also in temperate lands of Europe, India, Pakistan, and Iran [1–3]. The plant is used for multiple purposes in traditional medicines, like sedative, hypnotic, expectorant, cough suppressant, purgative, demulcent, diuretic, antiseptic, vasorelaxant, and antispasmodic and hence used to manage bronchitis, asthma, pertussis, gastrointestinal, and various other ailments [1, 2]. A brown viscid substance obtained following evaporation of the plant juice, called lactucarium, contains lactucone, lactucin, and lactucic acids [3]. The plant contains vitamins, beta carotene, and iron [4]. The phytochemical investigations of seeds revealed the presence of alkaloids, the bitter substance lettuce, oxalic acid, lactucopicrin [1], and sesquiterpene esters [5]. The alkaloid, lactucin, isolated from the seeds, exhibited antipyretic activity [6], and a triterpenoid saponin isolated from stem possesses antibacterial activity [7]. The pharmacological investigations on plant revealed its analgesic, anti-inflammatory [8], and antioxidant activities due to high total phenolic contents which showed efficient free radical scavenging potential like quercetin [9–11]. 

Although *Lactuca serriola* has traditionally been used to manage intestinal, respiratory, and vascular ailments, scientific investigations to validate such uses are so far lacking. The present study was undertaken to explore the pharmacological potential of  70% aqueous methanol extract of the plant and to study its possible mechanism(s) of action. 

## 2. Materials and Methods

### 2.1. Plant Material

The aerial parts of *Lactuca serriola* were collected from the botanical garden of Bahaudin Zakariya University, Multan, in July, 2010. The plant was identified by the taxonomist, Professor Dr. Altaf Ahmad Dasti, at the Institute of Pure and Applied Biology, Bahauddin Zakariya University, Multan. A voucher specimen of the plant, labelled as P.Fl. 760-7, was deposited in herbarium of the same Institute. 

### 2.2. Extraction

The plant material was subjected to shade drying and rendered free of foreign material through manual picking and grinded with the help of a herbal grinder to a coarse powder, after being stored in air-tight jars till extraction. One kg of the powdered plant material was soaked in 70% aqueous methanol in an amber glass container at 25°C for 3 days with occasional shaking thrice a day. The soaked material was passed through a muslin cloth to remove the vegetative debris and the filtrate obtained was passed through a Whatman-1 filter paper. The filtrate was stored in amber glass air-tight container. The previously mentioned extraction procedure was subsequently repeated twice and filtrates of these three macerations were combined. The filtrate was subjected to evaporation at 37°C under reduced pressure on a rotary evaporator (Rotavapor, Buchi Labortechnik AG, Model 9230, Switzerland) attached with a vacuum pump and a recirculation chiller. A deep green extract was obtained in 18% yield, then stored at −20°C in amber colored jars. 

### 2.3. Chemicals and Drugs

All the chemicals, solvents, and drugs used were of reagent analytical grade. Acetylcholine chloride, atropine sulfate, carbachol, dicyclomine, dimethylsulfoxide, ethylenediamine tetraacetic acid, glucose, magnesium chloride, magnesium sulfate, phenylephrine, potassium chloride, potassium dihydrogenophosphate, sodium chloride, sodium bicarbonate, and sodium dihydrogenophosphate were purchased from Sigma Chemical Company, St. Louis, MO, USA. The calcium chloride was purchased from Merck (Merck, Darmstadt, Germany). 

### 2.4. Preparation of Plant Extract

The methanol extract was solubilized in dimethylsulfoxide (1% w/v solution) in a stock solution of 300 mg/mL, which was then diluted to 30.0 and 3.0 mg/mL. The dimethylsulfoxide alone did not show any biological activity. The physiological salt solutions were prepared fresh on the day of experiment in distilled water. 

### 2.5. Animals

Adult albino rabbits (1.0–1.5 kg) of either sex, purchased from the local market with age limit between 6-7 months, were used for the experiments. Animals were provided with fresh green fodder and tap water *ad libitum* and maintained in air conditioned room (23–25°C) at Faculty of Pharmacy, Bahauddin Zakariya University, Multan. All rabbits were kept in fasting condition for at least 24 hours before the commencement of experiments but had free access to water. The experiments were complied with the rulings of the Institute of Laboratory Animals Resources, Commission on Life Sciences, National Research Council [12] and approved by the Ethical Committee of the Bahauddin Zakariya University, Multan, with having reference number EC/12/2008 dated 15 October 2008.

### 2.6. Isolated Rabbit Jejunum Preparations

The rabbit was starved overnight and was sacrificed subsequent to a blow on the head. The abdomen was opened and jejunum was dissected out and cut to segments of about 2 cm in length following removal of adhering mesenteries. The segments were mounted between two stainless steel hooks in a 10 mL tissue bath, containing normal Tyrodes solution (pH 7.4), maintained at 37°C and aerated with carbogen (5% CO_2 _+ 95% O_2_). A preload of 1 g was applied and the tissue was allowed to equilibrate for a period of 30 min during which the tissue was washed with fresh fluid at an interval of every 10 min prior to exposure to any test material. The spontaneous contractions were recorded isotonically through a Power Lab Data Acquisition System (AD Instruments, Sydney, Australia) [13]. The rabbit jejunum exhibits rhythmic spontaneous contractions, allowing the testing for relaxant and/or contractile activities. The contractile activities of test materials on isolated rabbit jejunum preparations are comparable to that of carbachol (CCh), which is likely to be minimized by pretreatment with atropine (0.1 *μ*M). The dose response curves for CCh in the presence of different concentrations of the methanol extract were then plotted using Computer Software GraphPad Prism.

### 2.7. Determination of Ca^++^ Channel Blocking Activity

The Ca^++^ channel blocking activity was determined by application of the methanol extract on K^+^-(80 mM) induced spastic contractions in isolated rabbit jejunum preparations [14]. The isolated rabbit jejunum preparations after exposure to K^+^ (80 mM) exhibited a sustained contraction, and the material was tested by its addition to the isolated tissue bath, in a cumulative manner to demonstrate concentration-dependant relaxant effects in the isolated tissue preparation [15]. The K^+^-(80 mM) induced smooth muscle contraction has been reported to be mediated through influx of Ca^++^ from extracellular fluid, and the substances capable to inhibit such contractions are speculated to be acting through the blockade of Ca^++^ channels [16]. The Ca^++^ channel blocking effect of the test material was further confirmed by the previously reported method [13]. The isolated rabbit jejunum preparations were allowed to be stabilized in normal Tyrode's solution, which was subsequently substituted with K^+^-normal but Ca^++^-free Tyrode's solution for 45 min, to which EDTA (0.1 mM) was already been added to remove Ca^++^ from the tissues. The solution was further replaced with K^+^-rich and Ca^++^-free Tyrode's solution having the following composition (mM): KCl (50), NaCl (91.04), MgCl_2_ (1.05), NaHCO_3_ (11.90), NaH_2_PO_4_ (0.42), glucose (5.55), and EDTA (0.1). After an incubation period of 30 min, the Ca^++^ was added to tissue bath in a cumulative manner to obtain control Ca^++^ dose-response curves (CDRCs). The stepwise increase in contractile activity of the tissue indicated that the extent of contractions was dependant on the availability of extracellular Ca^++^ for K^+^-induced influx of Ca^++^. When successive CDRCs for Ca^++^ were found to be superimposable, the tissue was washed and was allowed to be equilibrated with the methanol extract for 60 min, and then concentration response curves of Ca^++^ were reconstructed and compared to the CDRCs. The concentration response curves for Ca^++^ were developed in the presence of various concentrations of the methanol extract to assess a possible Ca^++^ channel blocking effect. The Ca^++^ channel blocking activity was also confirmed when the methanol extract caused shifting of the CDRCs constructed in calcium-free medium towards right in a concentration-dependant manner [16].

### 2.8. Isolated Rabbit Tracheal Preparations

The trachea was dissected out and cut into rings of 3-4 mm in width, each contains about two cartilages. Each ring was opened by a longitudinal cut on ventral side opposite to the smooth muscle layer, forming a tracheal strip with a central part of smooth muscle sandwiched between cartilaginous portions on the edges. The preparation was suspended in a 10 mL tissue bath containing Krebs physiological salt solution at 37°C and aerated with carbogen. About 1 g tension was applied to each of tracheal strips; this tension remained constant throughout the experiment. The isolated rabbit tracheal preparation was equilibrated for 45 min prior to recording isometric contractions via force displacement transducers connected to a Powerlab Data Acquisition System (AD Instruments, Sydney, Australia) and displayed on a computer running Lab Chart. The relaxant effect of the test material was assessed on carbachol-(1 *μ*M) and K^+^-(80 mM) induced contractions in isolated rabbit tracheal preparations as the cumulative addition of the test material to the isolated tissue bath may relax the isolated rabbit tracheal preparation. The isolated rabbit trachea preparations were equilibrated for 1 hr prior to the addition of any test substance. Carbachol (1 *μ*M) and K^+^ (80 mM) were used to produce sustained contractions in isolated rabbit trachea preparations, on which the possible bronchorelaxant activity of the methanol extract was studied following addition to the tissue baths in a cumulative manner in comparison to control drugs. The cumulative concentration response curves for carbachol were constructed through cumulative increase in concentration of agonist in tissue bath till a 3-fold increase in cumulative concentration did not produce further increase in response. The tissues were washed to reestablish the base-line tension, and CRCs for CCh were prepared in the presence of different concentrations of the methanol extract and the standard drug, dicyclomine [17]. 

### 2.9. Isolated Rabbit Aorta Preparations

The influence of the methanol extract on systemic vascular resistance was assessed on rabbit thoracic aorta as described previously [18]. The rabbit chest was opened; aorta was dissected out and kept in normal Krebs solution. The tissue was rendered free of connective tissues and loosely attached fats. The aortic rings of about 2-3 mm wide were mounted individually in 10 mL tissue bath containing Krebs solution at 37°C and aerated with carbogen. About 2 g preload was applied to each tissue preparation, and tissues were allowed to be equilibrated for a period of 1 hr before exposure to the test materials, during which Krebs solution was replaced after every 15 min. The vasoconstrictive effect of the methanol extract was assessed on cumulative addition of the test material to tissue organ bath, whereas vasorelaxant activity was noted on cumulative application to isolated tissue bath containing tissue preparation already contracted by phenylephrine (1 *μ*M) or K^+^ (80 mM). The changes in isometric tension of the aortic rings were recorded via a force-displacement transducer (model FORT100, WPI, USA) linked to a Powerlab Data Acquisition System (AD Instruments, Sydney, Australia) and displayed on a computer running Lab Chart software (version 6). 

### 2.10. Statistical Analysis

The results for spasmolytic, spasmogenic, bronchorelaxant, and vasorelaxant activities are expressed as the mean ± SEM. EC_50_ values with 95% confidence interval were calculated using the computer software GraphPad Prism program version 5.0 for Windows, (GraphPad, San Diego, USA). Dose-response curves were analyzed by nonlinear regression sigmoidal response curve (variable slope). 

## 3. Results

### 3.1. Effect on Isolated Rabbit Jejunum Preparations

**Figure 1:**
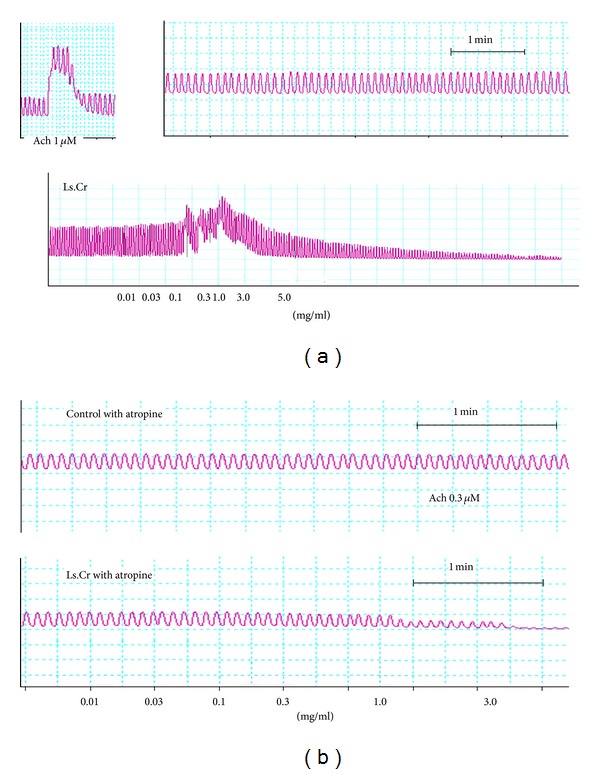
Effects of the methanol extract of *Lactuca serriola* (Ls. Cr) on spontaneously contracting isolated rabbit jejunum in the (a) absence and (b) presence of atropine.

**Figure 2:**
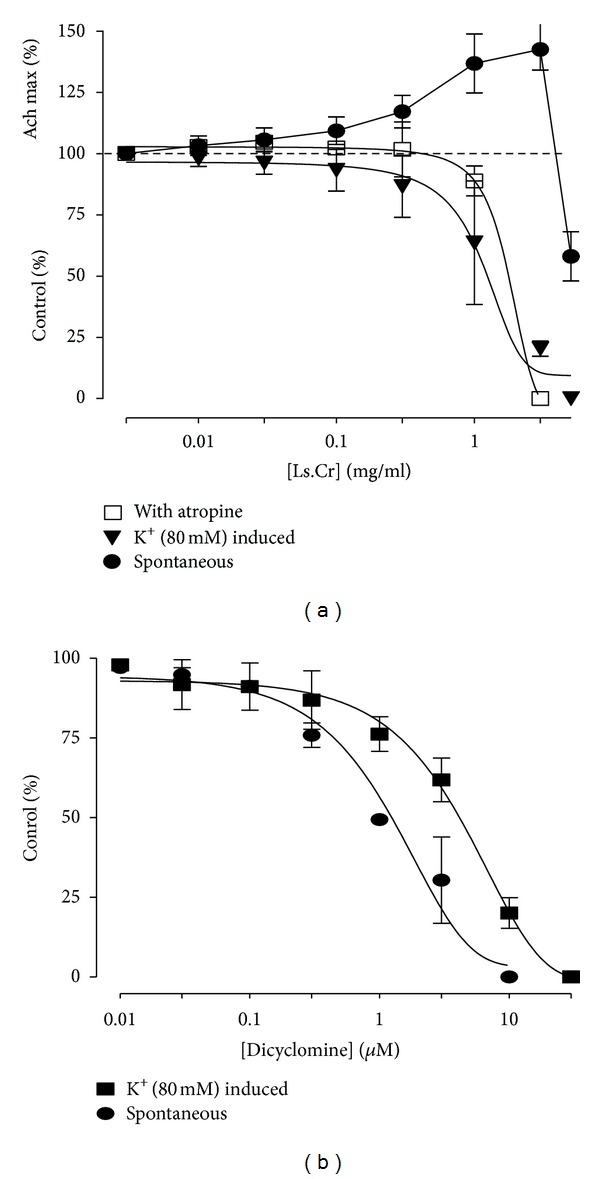
Concentration-dependent inhibitory effects of (a) methanol extract of *Lactuca serriola*, (b) dicyclomine on spontaneous and high K^+^-(80 mM) induced contractions in isolated rabbit jejunum preparation (values are expressed as the mean ± SEM; *n* = 5).

**Figure 3:**
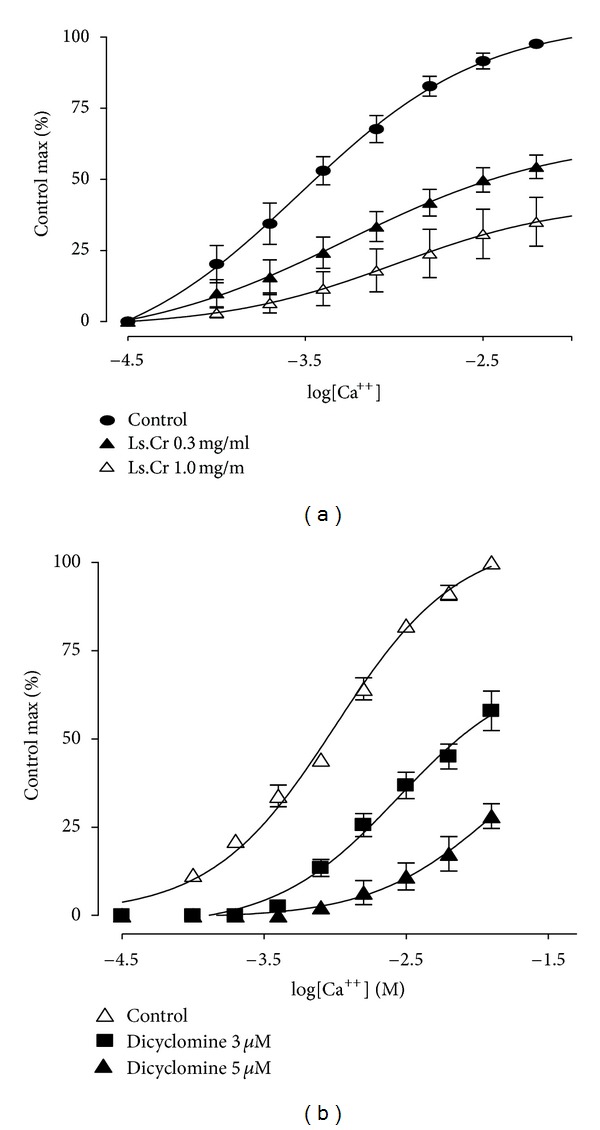
Concentration response curves of Ca^++^ in the absence (control) and presence of different concentrations of (a) methanol extract of *Lactuca serriola*, (b) dicyclomine*-*in isolated rabbit jejunum preparations (values are expressed as the mean ± SEM; *n* = 5).

The crude methanol extract of *Lactuca serriola* caused a concentration-dependent (0.03–3.0 mg/mL) contractile effect on the spontaneous contractions of isolated rabbit jejunum preparations, but it paradoxically showed a relaxant effect at higher concentration (5.0 mg/mL) and completely relaxed the tissue preparation (Figure 1(a)). However, the pretreatment of the tissue preparations with atropine (0.1 *μ*M) acaused suppression of the contractile response and relaxation of the spontaneous activity observed at a concentration of 3.0 mg/mL, with EC_50_ of 1.743 mg/mL (95% CI: 1.209–2.79; *n* = 5). This fact suggests a dual activity of the contractile response caused by the methanol extract, possibly mediated through muscarinic agonistic activity (Figure 1(b)). The complete relaxation of the K^+^-(80 mM) induced contractions was observed at tissue bath concentration of 5.0 mg/mL, with EC_50_ of 1.20 mg/mL (95% CI: 0.94–1.74: *n* = 5) (Figure 2). The standard drug, dicyclomine, exhibited a similar pattern of suppression of the spontaneous and K^+^-(80 mM) induced contractions with EC_50_ values of 1.15 *μ*M (95% CI: 0.87–1.53, *n* = 5) and 4.332 *μ*M (95% CI: 3.287–5.109, *n* = 5). The 45 min exposure to the methanol extract (0.3–1.0 mg/mL) of isolated rabbit jejunum preparations resulted in shifting the calcium dose response curves towards right, in a manner comparable to the effects of dicyclomine (3–5 *μ*M) (Figure 3).

### 3.2. Effect on Isolated Rabbit Tracheal Preparations

**Figure 4:**
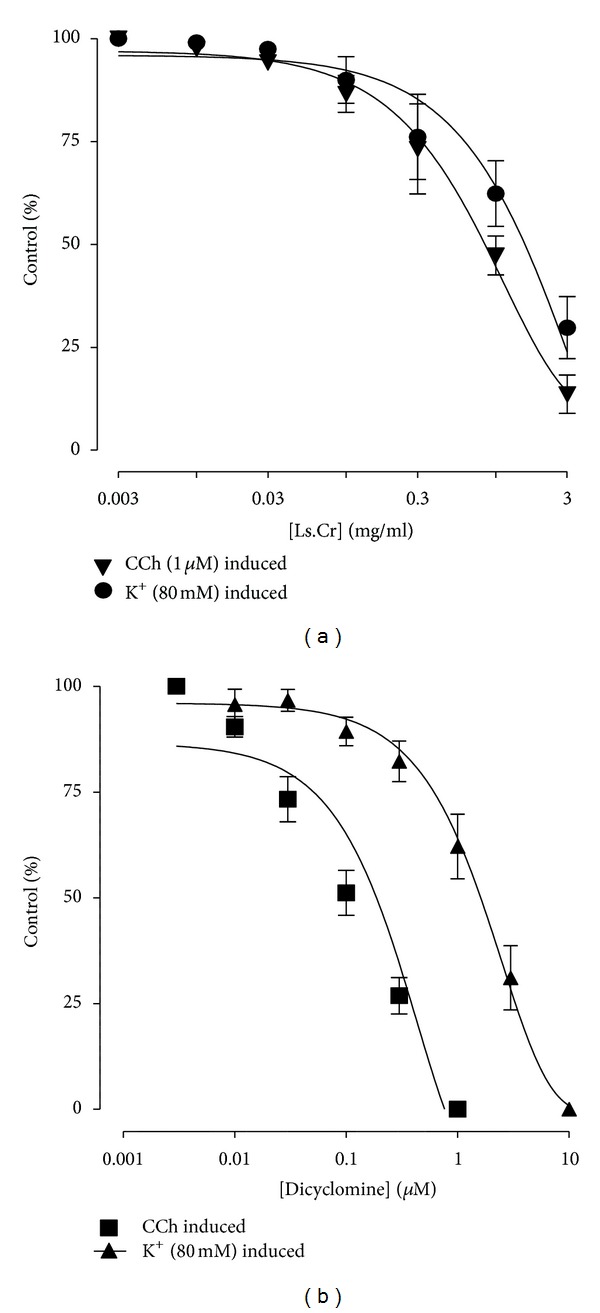
Concentration response curves showing the inhibitory effect of (a) methanol extract of *Lactuca serriola*, (b) dicyclomine on spontaneous and K^+^ (80 mM) and carbachol-induced contractions in isolated rabbit trachea preparations (values are expressed as the mean ± SEM; *n* = 5).

**Figure 5:**
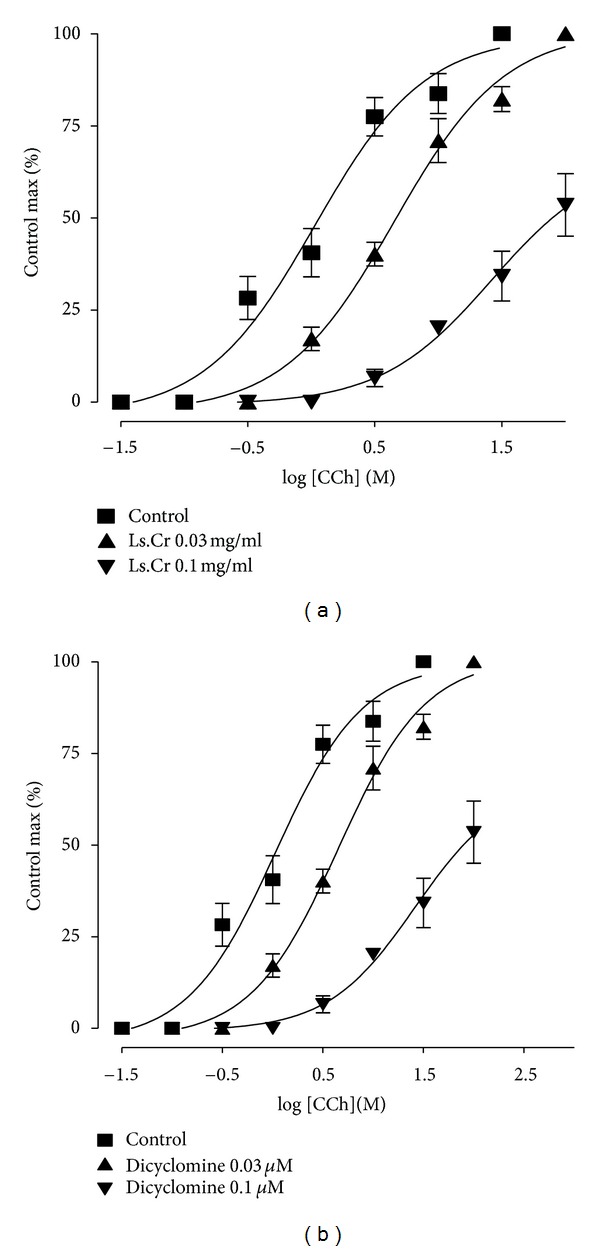
Concentration response curves of carbachol in the absence and presence of increasing concentrations of (a) methanol extract of *Lactuca serriola*, (b) dicyclomine in isolated rabbit trachea (values are expressed as the mean ± SEM; *n* = 5).

Concentration-dependant relaxant effect of methanol extract was observed when tested on carbachol-(1 *μ*M) and K^+^-(80 mM) induced contractions in isolated rabbit tracheal preparation at dose ranges of 0.01–1.0 mg/mL and 0.01–3.0 mg/mL, with EC_50_ values of 1.214 mg/mL (95% CI: 0.84–1.73; *n* = 5) and 2.11 mg/mL (95% CI: 1.53–2.90; *n* = 5), respectively (Figure 4). Similarly, dicyclomine caused relaxation of carbachol-(1 *μ*M) and K^+^-(80 mM) induced contractions with respective EC_50_ values of 0.171 *μ*M (95% CI: 0.102–0.220, *n* = 5) and 1.55 *μ*M (95% CI: 1.39–2.54, *n* = 5). The methanol extract at a concentration of 0.03 mg/mL caused rightward parallel shifting of the carbachol response curves without suppression of the maximum contractile response. However, a nonparallel shift was observed with suppression of maximum effect at a tissue bath concentration of 0.1 mg/mL, in a manner comparable to dicyclomine (0.03–0.1 *μ*M) (Figure 5).

### 3.3. Effect on Isolated Rabbit Aorta Preparations

**Figure 6:**
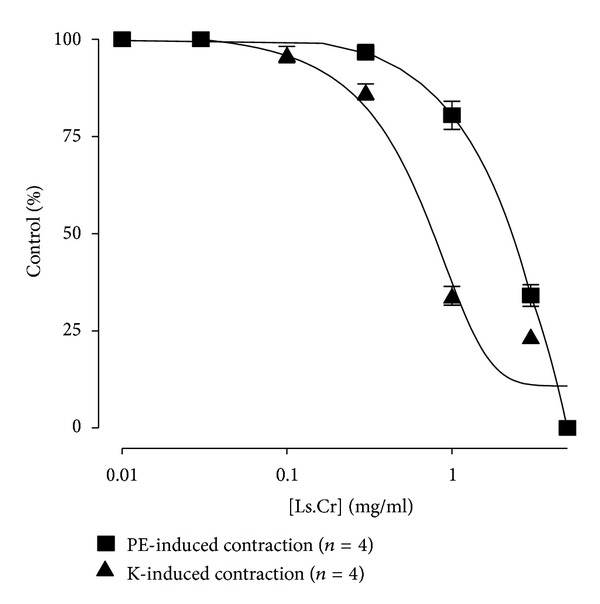
Concentration-dependent relaxant effect of methanol extract of *Lactuca serriola* on phenylephrine-(PE; 1 *μ*M) and K^+^-(80 mM) induced contraction in isolated rabbit aorta preparations (values are expressed as the mean ± SEM; *n* = 4).

The methanol extract, when tested on phenylephrine (1 *μ*M) and K^+^-(80 mM) induced contractions in isolated rabbit aorta preparations, caused concentration-dependent relaxation at the respective concentrations ranges of 0.01–3 mg/mL and 0.01–1.0 mg/mL with EC_50_ values of 1.259 mg/mL (95% CI: 1.015–1.561; *n* = 4) and 0.971 (95% CI: 0.66–1.41; *n* = 4), respectively (Figure 6).

## 4. Discussion

*Lactuca serriola* has traditionally been used as a purgative, a demulcent and an antispasmodic, and the present study was undertaken to validate its traditional uses. A methanol extract applied to spontaneously contracting isolated rabbit jejunum preparations exhibited concentration-dependant contractile effects at low concentrations, whereas it demonstrated relaxant effects at higher tissue bath concentration, indicating the presence of both gut stimulant and inhibitory constituents. The contractile response provoked by the methanol extract was comparable to that of acetylcholine, probably due to the presence of some cholinergic component(s). The acetylcholine-like mechanism of the contractile effect of the methanol extract of *L. serriola* was worked out after pretreatment of the isolated rabbit jejunum preparations with atropine (0.1 *μ*M), and the result was the suppression of the contractile effect and the domination of the relaxant effect. Acetylcholine is one of the neurotransmitters of autonomic nervous system, which through activation of M_3_ receptors can exert contractile activity in gut and is liable to be blocked by atropine, being one of the muscarinic antagonists [17, 19], and the role of acetylcholine in regulation of the peristaltic movements of gut has been documented [20]. The observed contractile effect of the methanol extract of *Lactuca serriola* in a manner similar to acetylcholine may provide a basis for the traditional use of the plant in constipation.

 The presence of calcium channel blocking activity may result in spasmolytic effects [21, 22]. The increase in free cytoplasmic Ca^++^ concentration causes activation of contractile element in smooth muscle preparations including that of rabbit jejunum [23, 24]. The intracellular Ca^++^ level is increased either by influx through the voltage-dependant Ca^++^ channels (VDCs) or release of Ca^++^ from intracellular stores in sarcoplasmic reticulum [25, 26]. The spontaneous movement of the intestine is mediated through periodic depolarization and repolarization; and in state of maximum depolarization, the rapid influx of Ca^++^ through VDCs is responsible for the appearance of a potential of action [27]. Thus, the relaxant effect of the methanol extract of  *L. serriola* observed on the hyperactive smooth muscle preparation may possibly be mediated either through blockade of VDCs or through inhibition of Ca^++^ released from sarcoplasmic reticulum. The exposure of isolated tissue preparations to high concentration of K^+^ (>30 mM) brings about opening VDCs, leading to influx of extracellular Ca^++^ and resulted in smooth muscles contraction [15, 24]. It can be hypothesized that the substances capable to inhibit K^+^-induced contractions are calcium channel blockers (CCBs). The extract was tested on K^+^-(80 mM) induced contractions to determine the possible mechanism of its observed antispasmodic effect. The isolated tissues exposed to K^+^ (80 mM) may result in depolarization of tissue, and the addition of the methanol extract to the isolated tissue baths caused concentration-dependant relaxation of K^+^-induced precontracted isolated jejunum preparation, due to decrease in Ca^++^ entry via VDCs. The previously-mentioned findings were confirmed further as the extract treatment of isolated tissues preparations resulted in decrease in tissue response to CaCl_2_ and rightward shifting in the concentration response curves of CaCl_2_, in a manner similar to that of dicyclomine, with dual action, that is, muscarinic receptors antagonist as well as blocker to Ca^++^ influx [28, 29]. Calcium channel blockers are reported to be effective in hyperactive gut diseases like diarrhea and abdominal cramps [30], and the calcium channel blocking activity in *L. serriola* methanol extract may provide a possible explanation of the traditional use of the plant in hyperactive disease states of the gastrointestinal tract, that is, diarrhea. 

*Lactuca serriola* has traditionally been used for the relief of multiple respiratory disorders including asthma, bronchitis, cough, and airway congestion. The possible bronchodilator activity of the crude extract of the plant was tested on carbachol-(1 *μ*M) and K^+^-(80 mM) induced sustained contractions on isolated rabbit tracheal preparations. The extract exhibited relaxant effect on both induced contractions, but CCh-induced contractions were found to be relaxed at much lower concentration in comparison to K^+^-(80 mM) induced contractions, in a manner similar to that of dicyclomine. Moreover, CCh is a cholinergic agonist which causes smooth muscle contraction through activation of muscarinic receptors [31]. Hence, the relaxation of airway muscles after the administration of the methanol extract was found to be due to the dual mechanism (i.e., muscarinic antagonist and Ca^++^ channel blockade). The bronchodilator effect may possibly be mediated through Ca^++^ channel blockade [32]. Interestingly, muscarinic antagonists are today used in the treatment for the relief from asthma and similar diseases [33]. The tone of bronchiolar smooth muscles is regulated by the parasympathetic division of the autonomic nervous system, and the reflex increase in parasympathetic activity may contribute towards bronchoconstriction, because respiratory tract is rich in cholinergic innervations through vagal fibres linked to M_1_ muscarinic receptors situated in the mucosal surface of the respiratory tract. The mucus secretions are also involved in adding up miseries to the pathology of the respiratory tract. In particular, submucosal glands are rich in parasympathetic innervations mostly through M_3_ receptors, and this may be one of the plausible explanations for using muscarinic antagonist in chronic obstructive pulmonary disease as well as asthma [34]. These results were confirmed further as the methanol extract of *L. serriola*, at a low tissue bath concentration of 0.03 mg/mL, displaced the CCh-concentration response curves in the isolated rabbit tracheal preparations to the right, in a parallel fashion without suppression of the maximum response. Moreover, following an increase in tissue bath concentration to 0.1 mg/mL, the log concentration response curve of carbachol was shifted to right in non-parallel manner with the suppression of the maximum response. The parallel shift of CCh-concentration response curves at low tissue bath concentration of the extract without suppression of maximal response can be an indication of antagonism of muscarinic receptors in competitive manner, whereas the nonparallel shift of CCh-concentration response curves at high tissue bath concentration with suppression of maximum response can be attributed to the presence of some components capable to exert Ca^++^ channel blocking effect. Interestingly, Ca^++^ channel blockers are known to be useful as tracheal relaxants in disorders characterized by hyperresponsiveness of respiratory tract [35, 36]. These findings are supportive for the traditional use of *Lactuca serriola* in respiratory ailments including asthma, bronchitis, cough, and respiratory congestion. 

The experimental findings on the methanol extract of *Lactuca serriola* suggested the presence of Ca^++^ channel blockers activity among the plant constituents, and Ca^++^ channel blockers have already been used in cardiovascular disorders [25, 26]. In order to assess the effect of the methanol extract on cardivascular system, it was applied to phenylephrine- and K^+^-(80 mM) induced contractions in rabbit aortic strips to distinguish between receptor mediated activity and blockade of L-type voltage-dependant Ca^++^ channels (VDCs). The exposition of rabbit aortic strips to K^+^ (80 mM) provoked smooth muscle contraction via activation of VDCs and the resultant release of calcium from sarcoplasmic reticulum [37]. On the other hand, phenylephrine causes increase in contractions of vascular smooth muscles through increased systolic Ca^++^ influx by two possible mechanisms, that is, influx of Ca^++^ via receptor operated channels and through release from intracellular stored calcium [38], thus signifying the blockade of Ca^++^ influx through receptor operated calcium channels [39]. In both cases, the net effect is the increased intracellular Ca^++^ concentration resulting in increased interaction between actin and myosin leading to contractile response. The methanol extract of *Lactuca serriola* was found to relax both phenylephrine- and K^+^-(80 mM) induced contractions in the rabbit aorta, suggesting that it may contain some constituents being active through both VDCs- and ROCs-mediated mechanisms.

## 5. Conclusion

The methanol extract of *Lactuca serriola* was found to possess spasmogenic, spasmolytic, bronchodilator, and vasorelaxant activities. The spasmogenic activity may be attributed to some cholinergic constituents, whereas spasmolytic effect may be due to Ca^++^ channel blocking components that may cause relaxation of gastrointestinal, tracheal, and aortic smooth muscles. The study may provide a scientific basis to validate the traditional use of the plant in the management of some gastrointestinal, respiratory, and vasospastic ailments.
